# PDIviz: analysis and visualization of protein–DNA binding interfaces

**DOI:** 10.1093/bioinformatics/btv203

**Published:** 2015-04-16

**Authors:** Judemir Ribeiro, Francisco Melo, Andreas Schüller

**Affiliations:** Departamento de Genética Molecular y Microbiología, Facultad de Ciencias Biológicas, Pontificia Universidad Católica de Chile, Santiago, Chile

## Abstract

**Summary: **Specific recognition of DNA by proteins is a crucial step of many biological processes. PDIviz is a plugin for the PyMOL molecular visualization system that analyzes protein–DNA binding interfaces by comparing the solvent accessible surface area of the complex against the free protein and free DNA. The plugin provides three distinct three-dimensional visualization modes to highlight interactions with DNA bases and backbone, major and minor groove, and with atoms of different pharmacophoric type (hydrogen bond donors/acceptors, hydrophobic and thymine methyl). Each mode comes in three styles to focus the visual analysis on the protein or DNA side of the interface, or on the nucleotide sequence. PDIviz allows for the generation of publication quality images, all calculated data can be written to disk, and a command line interface is provided for automating tasks. The plugin may be helpful for the detailed identification of regions involved in DNA base and shape readout, and can be particularly useful in rapidly pinpointing the overall mode of interaction.

**Availability and implementation:** Freely available at http://melolab.org/pdiviz/ as a PyMOL plugin. Tested with incentive, educational, and open source versions of PyMOL on Windows, Mac and Linux systems.

**Contact:** aschueller@bio.puc.cl

**Supplementary Information:** Supplementary data are available at *Bioinformatics* online.

## 1 Introduction

Specific recognition of DNA by proteins is a prerequisite of many biological processes and is essentially understood when analyzed at a three-dimensional (3D) structural level. The steadily increasing number of protein–nucleic acid complexes deposited in the Protein Data Bank (>4500 by end of 2014; Berman *et al.*, 2000) now allows for a more fine-grained structural analysis of the key determinants of protein–DNA interactions. These interactions may be broadly classified into base readout (sequence-dependent interactions with DNA bases via the major or minor groove) and shape readout (detection of global or local variation of the canonical DNA shape; Rohs *et al.*, 2010). However, protein–DNA interactions are complex and a simple recognition code does likely not exist (Slattery *et al*., 2014). 3D visual analysis of protein–DNA complexes today often requires the use of several computer programs for contact analysis and visualization. Here we present PDIviz, a plugin for the PyMOL molecular visualization system (Schrödinger, LLC) that is specifically designed to visualize protein–DNA interfaces and to focus visual analysis on various aspects of protein–DNA interactions.

## 2 Implementation

Protein–DNA interface detection is based on the method of differential solvent accessible surface area estimation. The plugin estimates the solvent accessible surface area (SASA) with the PyMOL command *get_area* (solvent radius: 1.4 Å), which employs the Shrake–Rupley algorithm (Shrake and Rupley, 1973). We compared the results against NACCESS, a reference software for SASA calculation (Hubbard and Thornton, 1993). To obtain surface areas comparable to NACCESS, the default van der Waals (vdW) radii in PyMOL were redefined according to the values published by Chothia (1975). We benchmarked PDIviz against NACCESS version 2.1.1 with a non-redundant set of 245 protein–DNA complexes derived from our Protein–DNA Interface Database (PDIdb; Norambuena and Melo, 2010) and obtained a low root mean squared difference of 0.078 Å^2^ per atom for SASA estimation (for details see Supplementary Material).

The basic approach of PDIviz is the calculation of various types of buried surface areas of the protein–DNA interface. First, a protein–DNA complex is loaded into PyMOL and the plugin calculates SASA of the complex, the free protein and the free DNA. To calculate the latter two surfaces the protein and DNA are each copied to a new molecular object (isolated), prior to SASA estimation. Second, the difference in SASA of the isolated protein and isolated DNA against the complex is calculated for each atom *i* according to Equation 1:

(1) $$\Delta SASA_{i}=SASA_{i}^{isolated}-SASA_{i}^{complex}$$

where ΔSASA is the buried surface area (BSA). PDIviz identifies surface areas interacting with different regions of DNA, namely DNA bases, the sugar-phosphate backbone, and the major and minor groove. The definition of major/minor groove atoms in duplex B-DNA is according to Seeman *et al.* (1976). To estimate BSA, the different DNA regions are first isolated. Next, BSA of protein atoms interacting with DNA bases is calculated as *SASA*(protein) – *SASA*(bases + protein); BSA of protein atoms interacting with DNA backbone is calculated as *SASA*(protein) – *SASA*(DNA backbone + protein); BSA of protein atoms interacting with the major groove of DNA is calculated as *SASA*(complex – major groove) – *SASA*(complex); and BSA of protein atoms interacting with the minor groove is calculated as *SASA*(complex – minor groove) – *SASA*(complex).

**Fig. 1.**
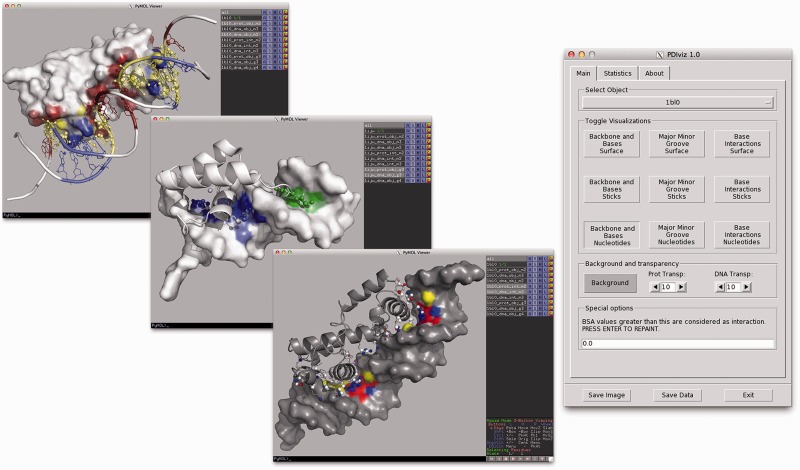
Protein-DNA interface visualizations created by PDIviz. The *MarA* protein-mar DNA complex (PDB code: 1BL0) is shown in the top panel on the left-hand side in the DNA backbone and base interactions mode in nucleotide style. The Hin DNA binding domain in complex with the *hixL* half-site (PDB code: 1IJW) is shown in the middle panel on the left-hand side in major/minor groove mode in surface representation. The potential pharmacophore interaction map of MarA-*mar* is shown in the lower panel on the left-hand side. The PDIviz graphical user interface window is shown on the right-hand side

PDIviz is easily installed via PyMOL’s Plugin Manager and started from the Plugin menu. The PDIviz plugin presents itself as a separate window with three different tabs (Fig 1): ‘Main’, containing the main controls for calculation and visualization; ‘Statistics’, containing a table listing calculated buried and accessible surface areas in Å^2^; and ‘About’, which contains a brief description of the plugin. Any protein–DNA complex loaded in PyMOL is automatically recognized by PDIviz and may be selected from the drop down box in the ‘Main’ tab. Calculations are executed by selecting any of the nine visualization modes. The BSA cutoff value may be configured by the user (default: >0.0 Å^2^), where higher values result in a smaller detected interface area. The PyMOL visualization window background color, and the protein and DNA surface transparency can also be modified to specific user needs. PDIviz provides three principal visualization modes, which highlight (i) interactions with DNA bases and the sugar-phosphate backbone (buttons of the 1st column), (ii) interactions with the major and minor groove (2nd column) and (iii) interactions with atoms of different pharmacophoric type (3rd column). In general, all atoms and surface areas involved in the protein–DNA interface are colored, while the remaining atoms and areas are shown in white. Color intensity (a color gradient blending into white) correlates with BSA. In the first mode, the sugar-phosphate backbone interface (DNA backbone atoms and protein atoms interacting with them) is colored in red, the DNA bases interface is colored in blue, and atoms/areas involved in simultaneous interaction with DNA bases and backbone are colored in yellow. In the second mode, the major groove interface is shown in blue, the minor groove interface is colored in green and simultaneous interaction with both grooves is shown in violet (possible in rare cases, e.g. damaged DNA). In the last mode (pharmacophore mode), hydrogen bond (H-bond) donors are shown in blue, H-bond acceptors are shown in red, donor/acceptor atoms (e.g. hydroxyl group) are colored in pink, thymine methyl groups are shown in yellow and other interface atoms are colored white. Here, all remaining non-interface atoms are colored in gray, according to the color scheme published by Rohs *et al.* (2010). Each visualization mode additionally comes in three different styles. Buttons of the first row show the DNA in surface mode and the protein in balls-and-sticks mode, thus focusing on the protein side of the interface. All atoms involved in interactions are shown as balls, while other atoms of interface residues are shown as sticks. Buttons of the second row focus on the DNA side: they show the protein in surface mode and the DNA as balls and sticks. The last row of buttons is a variation of the previous mode (protein as surface, DNA as balls and sticks). However, this mode focuses on the nucleotide sequence. If a nucleotide contains at least one atom participating in the protein–DNA interface, the entire nucleotide is colored according to the color scheme defined above. Since entire nucleotides are colored, continuous sequence stretches involved in certain types of interactions are easily identified in 3D and also at the sequence level with help of PyMOL’s sequence browser.

Finally, publication quality images may be saved via the ‘Save Image’ button and detailed tabular SASA and BSA data may be saved as text files with help of the ‘Save Data’ button. Advanced user may find the command line interface useful to run PDIviz in batch mode. A manual with a full description of these features is available with the software release from our website at http://melolab.org/pdiviz/.

In conclusion, PDIviz is a novel plugin for PyMOL that provides an intuitive way of visualizing protein–DNA binding interfaces detected by solvent accessible surface area estimation. Nine visualization modes are available, which help focusing on various aspects of protein–DNA interactions such as specific versus unspecific binding and base versus shape readout. Additionally, PDIviz may be useful in rapidly pinpointing the overall mode of interaction.

## Supplementary Material

Supplementary Data
