# Supplementary material for: PDIviz: analysis and visualization of protein–DNA binding interfaces
Source: Bioinformatics. 2015 Apr 16;31(16):2751–3. doi: 10.1093/bioinformatics/btv203 (PMC4528634; doi:10.1093/bioinformatics/btv203)
Supplement: Supplementary Data [file supp_31_16_2751__index.html]

PDIviz: analysis and visualization of protein–DNA binding interfaces — PDIviz: analysis and visualization of protein–DNA binding interfaces — Supplementary Data 

# PDIviz: analysis and visualization of protein–DNA binding interfaces

## Supplementary Data

files

**Files in this Data Supplement:**

- Supplementary Data - pdf file
