# Supplementary material for: PDIviz: analysis and visualization of protein–DNA binding interfaces
Source: Bioinformatics. 2015 Apr 16;31(16):2751–3. doi: 10.1093/bioinformatics/btv203 (PMC4528634; doi:10.1093/bioinformatics/btv203)
Supplement: Supplementary Data [file supp_btv203_btv203-Ribeiro_et_al_Suppl_Mat.pdf]

PDIviz: analysis and visualization of protein-DNA binding interfaces

Judemir Ribeiro, Francisco Melo and Andreas Schüller*

Departamento de Genética Molecular y Microbiología, Facultad de Ciencias Biológicas, Pontificia Universidad Católica de Chile, Alameda 340, Santiago, Chile.

* To whom correspondence should be addressed. Contact: aschueller@bio.puc.cl

VALIDATION OF SASA CALCULATIONS

The solvent accessible surface area (SASA) of 245 non-redundant protein-DNA complexes was estimated with the command *get_area* of PyMOL (incentive version 1.7.4.1; Schrödinger, LLC) and with the software NACCESS (version 2.1.1; Hubbard and Thornton, 1993). The default van der Waals atom radii values of PyMOL were modified according to the values published by Chothia, 1975: oxygen 1.40 Å, trigonal nitrogen 1.65 Å, tetrahedral nitrogen 1.50 Å, tetrahedral carbon 1.87 Å, trigonal carbon 1.76 Å, sulphur 1.85 Å and water 1.40 Å. These values correspond to the same radii utilized by the NACCESS program. A non-redundant set of 245 protein-DNA complexes was obtained from our Protein-DNA Interface Database (PDIdb; Norambuena and Melo, 2010). Briefly, the amino acid sequences of the protein chains of 922 protein-DNA interface complexes were clustered with the computer program BLASTClust (Altschul et al., 1990), according to a length coverage threshold of 90% and percentage sequence identity of 70%. The resulting set is non-redundant in terms of the protein sequences and is available from our web site at http://melolab.org/pdidb/. A single structure (PDB code: 1qpi) was excluded from the analysis due to errors in the PDB file. All PDB files were processed with a script to remove atoms/residues/chains with alternative locations (altloc), keeping the location of higher occupancy, or in case of same occupancy, keeping the first position in alphabetical order. PDIviz 1.0 (PyMOL) and NACCESS calculations were compared per atom (Fig. S1) and per protein-DNA complex structures (Fig. S2), and results are summarized in Table S1 (per atom) and Table S2 (per structure).

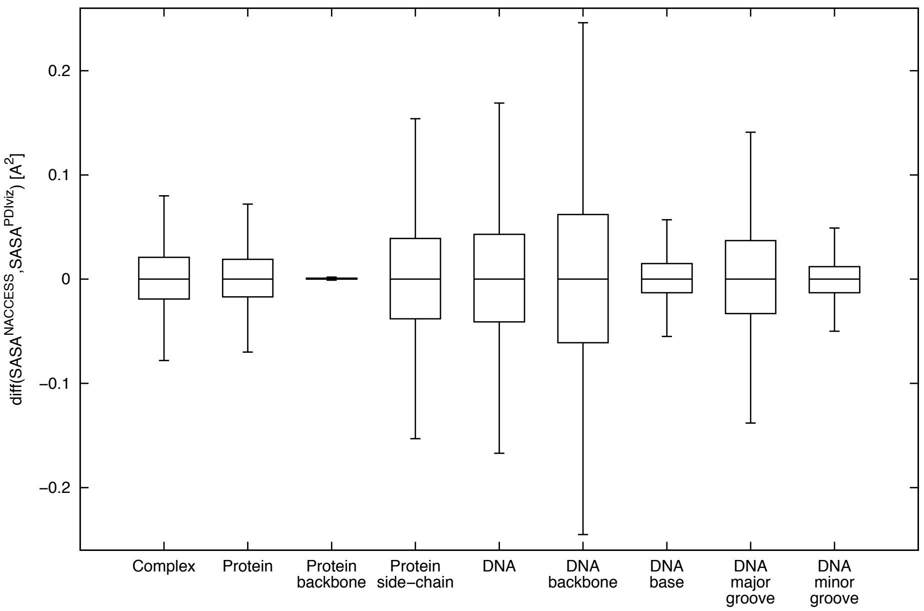

Fig. S1. Difference of estimated SASA per atom. SASA for all atoms of the non-redundant set was calculated with NACCESS 2.1.1 and PDIviz 1.0 (Incentive PyMOL 1.7.4.1). For each atom the difference of the accessible surface area estimations was calculated and plotted as box plots. Box boundaries represent the 1st and 3rd quartile and box centers indicate the median. Whiskers are drawn at 1.5 IQR (interquartile range).

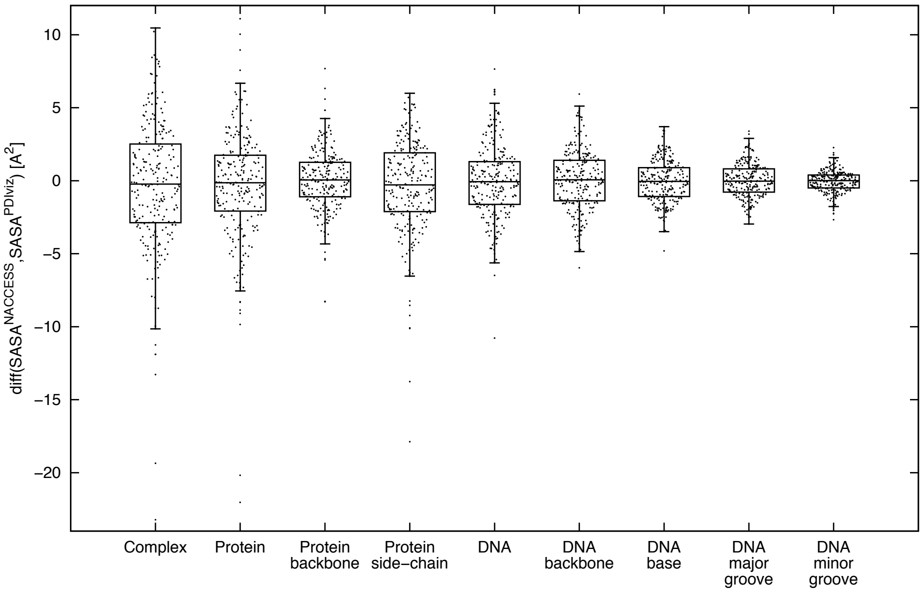

Fig S2. Difference of estimated SASA per structure. SASA for all structures (protein-DNA complexes) in the non-redundant set was calculated with NACCESS 2.1.1 and PDIviz 1.0 (Incentive PyMOL 1.7.4.1). For each structure the difference of the accessible surface area estimations was calculated and plotted as box plots. Box boundaries represent the 1st and 3rd quartile and box centers indicate the median. Whiskers are drawn at 1.5 IQR (interquartile range). Values are plotted as swarms of black dots.

Table S1. Differences of SASA estimations per atom.

	N	Quartile 1	Median	Quartile 3	Mean	Std. dev.	RMSD
Complex	756832	-0.019	0.000	0.021	0.000	0.078	0.078
Protein	567905	-0.017	0.000	0.019	0.000	0.079	0.079
Protein backbone	284645	0.000	0.000	0.001	0.000	0.057	0.057
Protein side chain	283260	-0.038	0.000	0.039	0.000	0.096	0.096
DNA	188927	-0.041	0.000	0.043	0.000	0.091	0.091
DNA backbone	100786	-0.061	0.000	0.062	0.000	0.105	0.105
DNA bases	88141	-0.013	0.000	0.015	0.000	0.072	0.072
DNA major groove	44173	-0.033	0.000	0.037	0.000	0.084	1.144
DNA minor groove	30273	-0.013	0.000	0.012	0.000	0.066	0.751

Differences are calculated as $Diff(SASA) = SASA_i^{NACCESS} - SASA_i^{PDIviz}$, where *Diff*(*SASA*) denotes the (possibly signed) difference of a SASA estimate of atom *i*. $RMSD = \sqrt{\sum_1^N (SASA_i^{NACCESS} - SASA_i^{PDIviz})^2 / N}$ denotes the root mean squared deviation of SASA estimates. N is the total number of differences and std. dev. refers to the standard deviation.

Table S2. Differences of SASA estimations per structure.

	N	Quartile 1	Median	Quartile 3	Mean	Std. dev.	RMSD
Complex	245	-2.879	-0.233	2.509	-0.324	4.516	4.519
Protein	245	-2.084	-0.138	1.748	-0.366	3.876	3.885
Protein backbone	245	-1.105	0.048	1.264	0.029	2.077	2.073
Protein side chain	245	-2.117	-0.285	1.910	-0.395	3.266	3.283
DNA	245	-1.626	-0.069	1.301	-0.001	2.566	2.561
DNA backbone	245	-1.371	0.060	1.396	0.057	2.099	2.096
DNA bases	245	-1.083	-0.051	0.898	-0.058	1.441	1.440
DNA major groove	245	-0.780	-0.032	0.807	0.023	1.146	1.144
DNA minor groove	245	-0.494	0.008	0.386	-0.058	0.750	0.751

Differences are calculated as $Diff(SASA) = SASA_i^{NACCESS} - SASA_i^{PDIviz}$, where *Diff*(*SASA*) denotes the (possibly signed) difference of a SASA estimate of structure *i*. $RMSD = \sqrt{\sum_1^N (SASA_i^{NACCESS} - SASA_i^{PDIviz})^2 / N}$ denotes the root mean squared deviation of SASA estimates. N is the total number of differences and std. dev. refers to the standard deviation.

REFERENCES

Altschul,S.F. et al. (1990) Basic local alignment search tool. *J Mol Biol* **215**, 403-410.

Chothia,C. (1975) Structural invariants in protein folding. *Nature* **254**, 304-308.

Hubbard,S.J. and Thornton,J.M. (1993) “NACCESS”, Computer Program, Department of Biochemistry and Molecular Biology, University College London.

Norambuena,T. and Melo,F. (2010) The Protein-DNA Interface database. *BMC Bioinformatics*, **11**, 262.
